# Can We End the Age-Old Problem of Pressure Injuries?

**DOI:** 10.1093/geroni/igaa057.2910

**Published:** 2020-12-16

**Authors:** Tracey Yap

**Affiliations:** Duke University, Chapel Hill, North Carolina, United States

## Abstract

A pressure injury/ulcer (PrI) is a localized area of injured skin and tissue usually over a bony prominence and one of the highest priority problems identified in U.S. health care’s federal quality initiatives; approximately 26.8 billion is spent for treatment each year. The problem is accentuated for nursing home residents who are often immobile/bedridden. Currently, resident repositioning/movement by nursing staff every 2-hours is the cornerstone of prevention care. Successful interventions must be nurse-led and designed to facilitate the prevention care of nursing staff on the front lines. My research focuses on integrating movement into everyday care for institutionalized older adults and is advancing the science of PrI prevention through testing of cueing interventions for nursing staff to improve the care delivery. My goals for innovating preventive care include enhancing our understanding of nursing subcultures’ influence on care outcomes and leveraging emerging technology to enhance the care team’s collaborative efforts.

